# Estimation of insecticide persistence, biological activity and mosquito resistance to PermaNet® 2 long-lasting insecticidal nets over three to 32 months of use in Ethiopia

**DOI:** 10.1186/1475-2875-13-80

**Published:** 2014-03-06

**Authors:** Gedeon Yohannes Anshebo, Patricia M Graves, Stephen C Smith, Aprielle B Wills, Mesele Damte, Tekola Endeshaw, Estifanos Biru Shargie, Teshome Gebre, Aryc W Mosher, Amy E Patterson, Paul M Emerson

**Affiliations:** 1 The Carter Center, Addis Ababa, Ethiopia; 2 Present address: School of Public Health, Tropical Medicine and Rehabilitation Sciences, James Cook University, PO Box 6811, Cairns, Qld, Australia; 3 The Carter Center, Atlanta, GA, USA; 4 Division of Parasitic Diseases and Malaria, Centers for Disease Control and Prevention, Atlanta, GA, USA; 5 Present address Abt Associates, Africa IRS, Addis Ababa, Ethiopia; 6 Present address: The Global Fund, Geneva, Switzerland; 7 Present address: International Trachoma Initiative, Addis Ababa, Ethiopia; 8 Present address: The Gates Foundation, Seattle, WA, USA; 9 Present address: Agnes Scott college, Decatur, GA, USA; 10 Present address: International Trachoma Initiative, Atlanta, GA, USA

## Abstract

**Background:**

Information is needed on the expected durability of insecticidal nets under operational conditions. The persistence of insecticidal efficacy is important to estimate the median serviceable life of nets under field conditions and to plan for net replacement.

**Methods:**

Deltamethrin residue levels were evaluated by the proxy method of X-ray fluorescence spectrometry on 189 nets used for three to six months from nine sites, 220 nets used for 14-20 months from 11 sites, and 200 nets used for 26-32 months from ten sites in Ethiopia. A random sample of 16.5-20% of nets from each time period (total 112 of 609 nets) were tested by bioassay with susceptible mosquitoes, and nets used for 14-20 months and 26-32 months were also tested with wild caught mosquitoes.

**Results:**

Mean insecticide levels estimated by X-ray fluorescence declined by 25.9% from baseline of 66.2 (SD 14.6) mg/m^2^ at three to six months to 44.1 (SD 21.2) mg/m^2^ at 14-20 months and by 30.8% to 41.1 (SD 18.9) mg/m^2^ at 26-32 months. More than 95% of nets retained greater than 10 mg/m2 of deltamethrin and over 79% had at least 25 mg/m2 at all time periods. By bioassay with susceptible *Anopheles*, mortality averaged 89.0% on 28 nets tested at three to six months, 93.3% on 44 nets at 14-20 months and 94.1% on 40 nets at 26-32 months. With wild caught mosquitoes, mortality averaged 85.4% (range 79.1 to 91.7%) at 14-20 months but had dropped significantly to 47.2% (39.8 to 54.7%) at 26-32 months.

**Conclusions:**

Insecticide residue level, as estimated by X-ray fluorescence, declined by about one third between three and six months and 14-20 months, but remained relatively stable and above minimum requirements thereafter up to 26-32 months. The insecticidal activity of PermaNet® 2.0 long-lasting insecticidal nets in the specified study area may be considered effective to susceptible mosquitoes at least for the duration indicated in this study (32 months). However, results indicated that resistance in the wild population is already rendering nets with optimum insecticide concentrations less effective in practice.

## Background

Insecticide-treated nets (ITNs) are one of the principal mainstays of malaria control efforts currently in use, with over 100 countries having adopted the WHO recommendation of universal coverage [1,2]. The percentage of households owning at least one ITN in sub-Saharan Africa is estimated to have risen from 3% in 2000 to 53% in 2012 [2]. This massive scale-up coincided with a significant drop in malaria incidence and mortality in the last decade. Maintaining the levels of household net ownership and ensuring that sufficient nets are provided for access to all household members requires a huge financial and logistical investment, with Africa alone needing approximately 150 million new ITNs each year [2]. Therefore, it is essential that information is gathered on the expected life of an ITN under actual usage in order to protect the massive investment in malaria vector control activities and plan effectively for timely net replacement.

Long-lasting insecticidal nets (LLINs) were developed to meet the need for ITNs that did not require frequent retreatment during their physical lifespan. Therefore, the ability of these products to meet minimum performance requirements over long periods of time and under actual usage conditions and at a reasonable cost should be the prime measure of their acceptability. Before the large-scale distribution of LLINs, performance was evaluated using laboratory testing (e g, wash-resistance experiments), experimental hut trials and limited field testing under controlled conditions [3,4].

Net durability comprises three aspects: physical durability, persistence of insecticide and ability of the nets to kill mosquitoes (which depends not only on the net, but the susceptibility status of the local mosquito population). Two methods are used for assessment of chemical (insecticide) durability and recommendation by the WHO Pesticide Evaluation Scheme (WHOPES) [5,6]: bioassay on nets using the cone method, and the tunnel test. Nets of any type must pass a bioassay criterion of >95% knockdown at 60 mins or > =80% mortality at 24 hours after a 3-minute exposure in the cone test, which is used here. For PermaNet 2.0, the specifications state that the concentration of active ingredient must be 1.8 and 1.4 g/kg, respectively for a 75 - and 100-denier yarn, and vary by plus or minus 25% or less. This corresponds to a target dose of 55 mg/m^2^ on 75-denier nets when the nets are new.

Scale-up of net distribution started in 2006 in Ethiopia and rapidly achieved a high level of net ownership [7,8]. Samples of nets with known batch numbers that were available for distribution in 2007, with assistance from The Carter Center, were collected at three time intervals over three to 32 months in 2007, 2008 and 2009 [9]. For this sample of nets, it was previously reported [9] that physical damage to nets starts early and accumulates rapidly. Using a proportionate hole index, more than 90% of nets had at least one hole and one third of nets were classified as unusable and ineffective after two and a half years in use [9].

The current study addresses the specific issue of insecticidal content of LLIN samples collected after a period of three to 32 months since distribution in Ethiopia in 2007, as described previously [9]. While there have been several studies on the physical durability of nets, relatively few [10-15] have tested the insecticidal content of nets used by householders under real-world conditions and over defined time periods. Kilian *et al*. [11] and Banek *et al.*[14] estimated that nets impregnated with alpha-cypermethrin lost insecticide at a rate of about 20% per year in the conditions studied. Kweka *et al.*[15] estimated the concentration of insecticide on nets when new and then five years later, but were only able to recover seven of the original nets distributed. They found that insecticide was undetectable after five years.

WHOPES recommends nets based on biological activity and insecticide persistence under laboratory and field conditions but not on measurable insecticide concentrations. The relationship between these two factors varies by the type of net and is not well defined, especially for LLINs where a large amount of the chemical is within the net fibres, from which it is slowly released towards the surface over time. Using conventionally treated nets, it was estimated that a concentration of 6-8 mg/m^2^ of deltamethrin was necessary to reach the WHOPES standard of 24-hr mortality in > =80% of mosquitoes (John Gimnig, pers comm). A similar estimate of minimum effective concentration was estimated by Kroeger *et al.*[16] for the first versions of PermaNet. An extensive study by Kilian *et al.*[17] showed that levels of 4 and 15 mg/m^2^ on both first and second generation PermaNets (assessed by gas chromatography) were associated with minimal and optimal effectiveness respectively against *Anopheles*, as defined by at least 90% of nets meeting the following criteria:

Minimal effectiveness: Knockdown at 60 minutes > =75% or mortality > =50%

Optimal effectiveness: Knockdown at 60 minutes > =95% or mortality > =80%.

There are two factors that suggest that 6-8 mg/m^2^ or even 15 mg/m^2^ are too low as concentration estimates for optimum effectiveness: the first is the fact that the gas chromatography method used by Kilian *et al.*[17] slightly underestimates deltamethrin (estimated by HPLC) by about 15%, and the second is that only a proportion of the measured insecticide in a LLIN is on the surface. A study by Green *et al.*[18] correlated surface concentration with high performance liquid chromatography (HPLC) estimates of total concentration of deltamethrin. Using a colorimetric assay for deltamethrin on the surface of PermaNets, it was observed that 80% killing activity was associated with a detected surface concentration of 2.8 ug/m^2^, but that this was equivalent to 20-25 mg/m^2^ of total concentration as detected by HPLC. Given this discrepancy, in this paper two concentrations levels of deltamethrin detected by X-ray fluorescence (XRF) are reported: 10 mg/m^2^ as a conservative estimate of minimum effective concentration, and 25 mg/m^2^ as a measure of optimum concentration. The bioassay results reported in this paper will contribute to further understanding of the relationship between deltamethrin concentration and mortality in exposed susceptible mosquitoes.

## Methods

### Net collections

**Figure 1:**
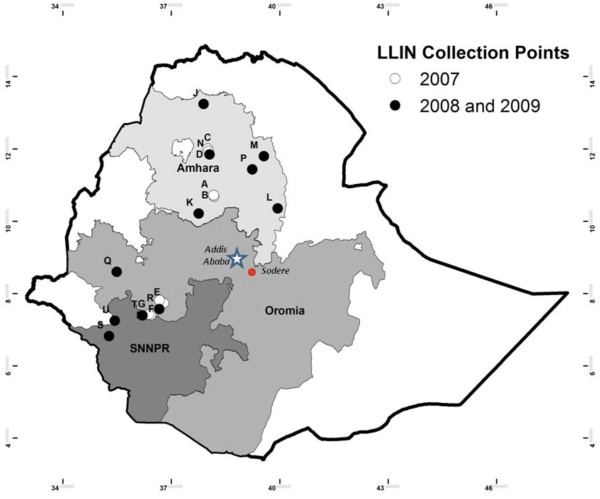
**Location of net collection sites A through U in Ethiopia.** Open circles denote areas of 2007-only collections. Closed circles denote areas where nets were recovered in both 2008 and 2009. Sodere denotes where larvae were collected to rear to adults for testing wild caught mosquitoes in bioassays.

Net distribution sites, collection procedure and numbers of nets collected were described previously [9]. Locations of sites are shown in Figure 1. In summary, 189 nets were collected from households in nine sites in 2007 and 2008 after three to six months in use; 220 nets were collected from 11 sites in 2008 and 2009 after 14-20 months in use, and 200 nets were collected from ten sites in 2009 after 26-32 months in use. The time of distribution was known from the batch number on the PermaNet 2 labels; other PermaNet 2 nets distributed at unknown times and found in households were collected in two sites in error but not analysed further once this was realized.

Nets from the first collection in 2007 were shipped to Atlanta, USA for testing. They were frozen at -80°C for three days before shipping to kill any bedbugs (bugs that may be found clinging to the nets). The second and third net collections in 2008 and 2009 were tested in Ethiopia.

A subset of 20% of the nets from each yearly batch collected was randomly selected (in Excel using the RAND function on records sorted by site and net number) for mosquito bioassays using a single 30 × 30 cm sample cut from one side of each of these nets. The samples from 32 nets randomly selected from sites A to H in the first collection in 2007 (after 3-6 months of use) were tested for insecticide by both XRF and HPLC after the mosquito bioassays were completed.

### High-performance liquid chromatography (HPLC)

For HPLC, a 30 × 30 cm sample cut from the side of each selected net was extracted in 100 ml of 77/23 acetonitrile/water for 1 hour in an ultrasonic bath. The HPLC analysis of deltamethrin concentration in the extract was carried out on an Agilent Series 1200 instrument equipped with a 125 × 4 mm Lichrospher 100 RP-18e 5 μm column using a 77/23 acetonitrile/water mobile phase and a flow rate of 1.0 ml/min at ambient temperature. Deltamethrin was detected using a UV detector set at 235 nm. For each sample, 5 injections of 20 μl each were made and compared with an external deltamethrin standard. After determining the concentration of deltamethrin in the extract, the concentration on the original 30 × 30 cm net sample was calculated.

### X-ray fluorescence analysis on single layers of netting

**Figure 2:**
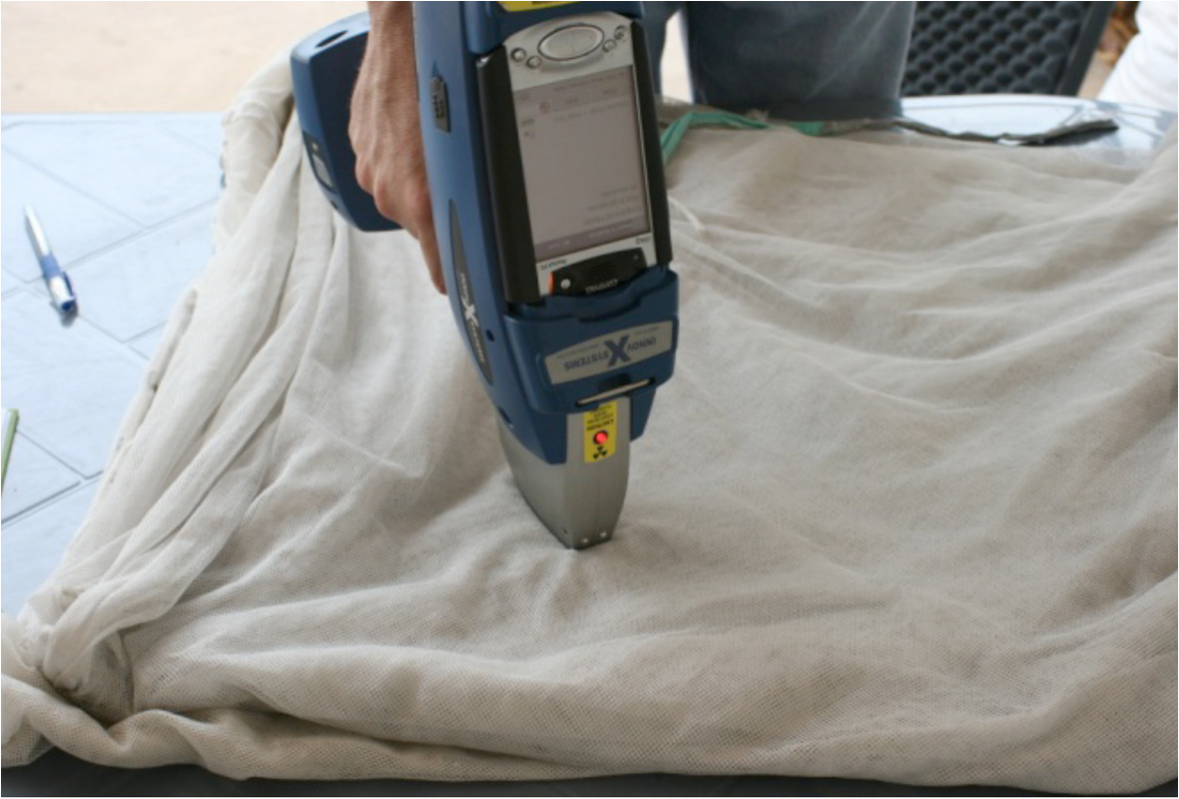
The X-ray fluorescence (XRF) detector used to estimate deltamethrin concentration on nets.

Each molecule of deltamethrin contains two bromine atoms. XRF analysis was conducted on each net to determine bromine content, using an Innov-X model XT-442 analyzer (Innov-X Systems, Woburn, MA, USA) (Figure 2) equipped with Empirical Mode v.1.45A software. Bromine content is assumed to correlate directly with concentration of the deltamethrin insecticide. The analyzer was initially calibrated using polyester netting treated with known concentrations of deltamethrin (K-O Tab™, Bayer Environmental Science, Monheim am Rhein, Germany). These calibration measurements were conducted on single layers of netting using a tin (Sn) plate background. The calibrated instrument was then used to measure deltamethrin content on single layers of PermaNet specimens previously recovered from the field, having a range of deltamethrin contents.

XRF readings were obtained from average of readings on single layers of net in the first collection in 2007, using five locations on each net: one on each side of the net, ascending from the bottom left corner to the upper right corner as the sampling progressed from one side to the next; and one additional reading from the top of the net. These readings were averaged to give the final XRF value for the net.

### X-ray fluorescence analysis on folded nets

**Figure 3:**
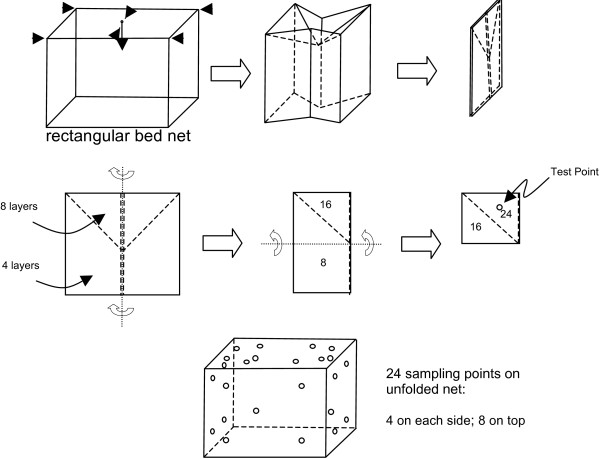
Method for folding net for X-ray fluorescence analysis.

For the bed nets collected from the second (2008) and third (2009) rounds of collections, deltamethrin content was determined by XRF analysis only. Nets were folded to present 24 layers of netting in a single reading (Figure 3). This allowed a mean deltamethrin concentration from all panels of the net to be taken in one measurement. Raw readings were divided by the number of layers (24) to produce the average deltamethrin concentration for a single layer of netting.

### Bioassays

Bioassays were performed on 30 × 30 cm pieces of a random sample of four nets from each collection site, except sites G and H at the first collection period (the randomly selected nets turned out not to be from The Carter Center (TCC) assisted distribution in 2007 and therefore of unknown age). A total of 28 nets were tested by bioassay after three to six months, 44 after 14-20 months, and 40 after 26-32 months.

In 2007, bioassays were done in Atlanta using the deltamethrin susceptible *Anopheles gambiae* Kisumu strain. Subsequent bioassays were done in Ethiopia at the Adama Malaria Training Centre against both the susceptible *Anopheles arabiensis* Nazareth strain and adults reared from wild caught *Anopheles* larvae from Sodere, Oromia region (Figure 1).

Bioassays used the Centers for Disease Control (CDC)-modified WHO cone method, with a three-minute exposure of 30-52 mosquitoes per sample in eight replicates, followed by a 24-hour holding period [5]. Knockdown at 30 and 60 minutes and mortality at 24 hours were assessed and adjusted for controls (untreated netting, run simultaneously) using Abbott’s formula [5].

### Statistical analysis

Findings from the two residue estimating techniques (XRF and HPLC) were compared using ordinary least squares regression analysis. Insecticide residue concentrations are described by the mean and standard deviation. Comparisons were done by t-test and analysis of variance with Bonferroni test for multiple comparisons. Proportions were compared by Chi-square tests and medians were compared by Mann-Whitney tests. Data management and analysis were done in Microsoft Excel, Access, SAS and STATA (versions 7 through 12).

## Results

### Validation of X-ray fluorescence as proxy for deltamethrin concentration

**Figure 4:**
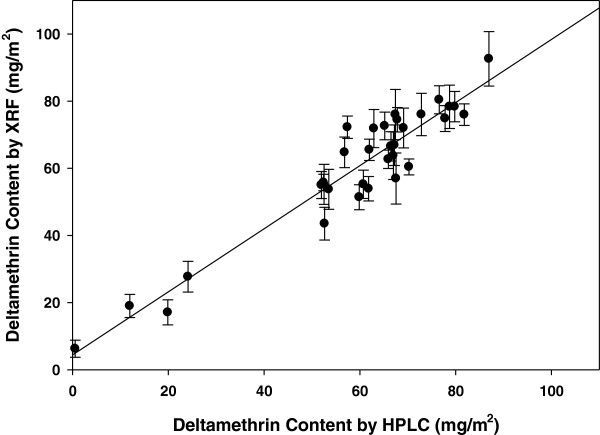
**Validation of XRF and HPLC methods on a subset of 32 nets collected after 3-6 months of use.** Error bars denote standard deviation. N = 5 readings for all points (sample of nets from each collection site in 2007).

A comparison of repeated XRF and HPLC measurement of deltamethrin levels on the 32 samples tested using both techniques is shown in Figure 4. Regression analysis showed a coefficient of 0.9397, with R^2^ = 0.9006, p = 0.001. Therefore, there is a strong relationship between XRF and deltamethrin concentration, with XRF slightly underestimating the true concentration. It is used here as a conservative and comparative method.

### XRF levels on all collected nets - by site and time period of collection

The mean concentrations of deltamethrin as measured by XRF on the samples of nets collected at each site and time period are shown in Table 1. The mean concentration of deltamethrin on the total sample of nets collected at three to six months was 66.2 mg/m^2^ (SD 14.6, N = 189); at 14-20 months it was 44.1 mg/m2 (SD 21.2, N = 220), and at 26-32 months was 41.1 (SD 18.9, N = 200). The difference between the three time groups of collection was significant by analysis of variance (F = 105.88, p < 0.001). The drop in mean insecticide concentration between three to six months and 14-20 months was highly significant (p < 0.001 by Bonferroni test), but there was little additional loss of insecticide between 14-20 and 26-32 months (p = 0.294).

**Table 1:** Insecticide levels of total sample of collected nets, by site and collection time

**Site**		**No of nets (no tested in bio-assay)**	**Deltamethrin concentration (mg/m**^**2**^**) (by XRF) Mean (SD)**	**No (%) of nets with <10 mg/m**^**2 **^**deltamethrin**	**Manufacturer’s reported batch mean deltamethrin concentration (MRBMC) (mg/m**^**2**^**)**	**Difference between field XRF and MRBMC (%)**
A		25 (4)	64.7 (13.3)	0	59.9	8.0
B		25 (4)	67.6 (10.8)	0	59.9	12.9
C		25 (4)	69.7 (14.3)	0	59.7	16.8
D		25 (4)	74.8 (12.5)	0	59.7	25.3
E		25 (4)	58.0 (14.3)	0	60.0	-3.3
F		25 (4)	58.1 (20.0)	1 (4)	60.0	-3.2
G		5 (0)	76.9 (5.6)	0	58.9	30.6
H		14 (0)	72.9 (9.5)	0	58.9	23.8
J		20 (4)	63.8 (8.6)	0	60.9	4.8
	***3–6 months***	***189 (28)***	***66.2 (14.6)***	***1 (0.5)***	59.8*	***10.8***
K		20 (4)	53.2 (15.9)	0	59.9	-11.2
L		20 (4)	38.1 (19.9)	1 (5)	59.1	-35.5
M		20 (4)	58.5 (14.7)	0	59.1	-1.0
N		20 (4)	41.6 (19.3)	1 (5)	59.7	-30.3
P		20 (4)	46.9 (19.9)	0	57.7	-18.7
Q		20 (4)	48.1 (19.0)	0	59.1	-18.6
R		20 (4)	30.7 (26.8)	6 (30)	60.0	-48.8
S		20 (4)	50.4 (26.0)	1 (5)	61.0	-17.4
T		20 (4)	47.1 (18.0)	0	58.9	-20.0
U		20 (4)	41.6 (19.3)	1 (5)	59.1	-29.6
J		20 (4)	28.7 (14.5)	2 (10)	60.9	-52.9
	***14-20 months***	***220 (44)***	***44.1 (21.2)***	***12 (5.5)***	59.5*	***-25.9***
K		20 (4)	40.0 (19.3)	2 (10)	59.9	-33.2
L		20 (4)	46.6 (13.9)	0	59.1	-21.2
M		20 (4)	54.3 (21.1)	0	59.1	-8.1
N		20 (4)	34.1 (14.7)	0	59.7	-42.9
P		20 (4)	35.5 (16.6)	1 (5)	57.7	-38.5
Q		20 (4)	42.0 (18.7)	0	59.1	-28.9
R		20 (4)	32.5 (18.0)	4 (20)	60.0	-45.8
S		20 (4)	45.3 (20.7)	0	61.0	-25.7
T		20 (4)	38.0 (14.0)	0	58.9	-35.5
U		20 (4)	42.4 (23.2)	1 (5)	59.1	-28.3
	***26-32 months***	***200 (40)***	***41.1 (18.9)***	***8 (4.0)***	59.4*	***-30.8***

^*^Average of the sites in the time group of collection.

There were ten batches of nets from different manufacturing runs included in this study. The WHOPES recommended target concentration on nets is 55 mg/m^2^, plus or minus 25% [6]. The overall mean concentration of deltamethrin on the nets when new, estimated by average of the manufacturer’s reported batch means, was 59.5 mg/m^2^, with a range between batches of 57.7 and 61.0 mg/m^2^. The estimated mean concentration of the ten batches when new was therefore slightly lower than that recorded by XRF on the used nets after three to six months in the field (66.2 mg/m^2^) (Table 1). However, the range of concentrations estimated by XRF on the individual nets at three to six months was very wide: 8.6 to 110.6 mg/m^2^, suggesting that some nets had much higher concentrations than the manufacturer’s specifications when distributed.

### XRF levels on all collected nets -- minimum effective concentration

After three to six months in the field, only 1/189 (0.5%) nets had an estimated concentration less than the proposed minimum effective concentration of 10 mg/m^2^ (Table 1). One additional net had less than suggested optimum concentration of 25 mg/m^2^ (overall two out of 189 = 1.1%). After 14-20 months the number of nets with <10 mg/m^2^ had increased to 12/220 (5.5%), significantly higher than the three to six-month nets (Chi^2^ = 8.01, p = 0.005). Similarly the number of nets with <25 mg/m^2^ had increased from 2/189 at three to six months to 49/220 or 22.3% at 14-20 months (Chi^2^ = 41.9, p < 0.001). At 26-32 months the proportion of nets with <10 mg/m^2^ was 8/200 (4.0%) and with <25 mg/m^2^ was 41/200 (20.5%), neither of which was significantly different from the 14-20 month group (Chi^2^ = 0.49, p = 0.484 and 0.20, p = 0.658).

**Figure 5:**
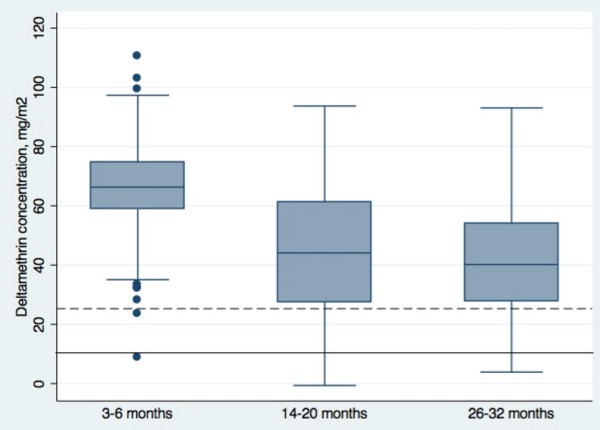
**Distribution of deltamethrin concentration on all collected nets, summarised by time group in potential use.** Box plots show the median, 25^th^ and 75^th^ percentiles (box) and fifth and 95^th^ percentiles (whiskers) for deltamethrin mg/m^2^ estimated by XRF. N = 189 for three to six month nets; N = 220 for 14-20 month nets; N = 200 for 26-32 month nets. The solid horizontal line shows minimum effective concentration at 10 mg/m^2^ and the dashed horizontal line shows optimum concentration at 25 mg/m^2^.

The distribution of deltamethrin concentrations on the complete sample of individual nets by time group (N = 189, 220 and 200 respectively) is shown in Figure 5, which illustrates the large range in amount of insecticide on the nets at three to six months, at both the high and low ends. Figure 5 also demonstrates the drop in median insecticide concentration between the first two time periods, with little further drop after 14-20 months. In addition, it shows that the 75% of the nets have insecticide concentrations above the optimum concentration of 25 mg/m^2^ in all groups, including the longest time period of 26-32 months.

### XRF levels and bioassay results on subsample of nets

Overall a sample of 18.4% of the nets (112/609) were tested by bioassay. Table 2 shows the deltamethrin X-ray measurement on each net that was tested by bioassay at three time periods. There was large variation between nets. After three to six months of use, the mean deltamethrin concentration was 61.3 mg/m^2^ (standard deviation (SD) 16.2 mg/m^2^). Only one of the 28 nets (3.6%) was below the minimum effective 10 mg/m^2^ concentration and no other nets were below 25 mg/m^2^. By 14 to 20 months (Table 2), the mean concentration had dropped to 42.8 mg/m^2^ SD 21.4 mg/m^2^). Two out of 44 tested nets (4.6%) were below 10 mg/m^2^ and a total of 12/44 (27.3%) were below 25 mg/m^2^. The drop in mean deltamethrin concentration for the bioassayed nets between the first two time periods was statistically significant (F = 15.36, p < 0.0002), as it had been for the total sample of all nets. At 26 to 32 months (Table 2), mean deltamethrin by XRF remained relatively stable at 45.1 mg/m^2^ (SD 19.1 mg/m^2^). This was not significantly different from the 14-20 month collection. None of the 40 nets tested at 26-32 months had a concentration below 10 mg/m^2^ but 7/40 (17.5%) were below 25 mg/m^2^.

**Table 2:** Bioassay results for tests on sample of collected nets, by site and collection time

			**Susceptible strain**						**Wild caught**					
**Site**	**No of nets**	**Mean (SD) deltamethrin concentration (mg/m**^**2 **^**by XRF)**	**Ave kd30 %**	**Range kd30 %**	**Ave kd60 %**	**Range kd60 %**	**Ave mort24h %**	**Range mort24h %**	**Ave kd30 %**	**Range kd30 %**	**Ave kd60 %**	**Range kd60 %**	**Ave mort24h %**	**Range mort24h %**
A	4	69.1 (8.8)	100	100–100	100	100–100	100	100–100						
B	4	71.9 (18.4)	100	100–100	100	100–100	100	100–100						
C	4	67.0 (7.5)	100	100–100	100	100–100	100	100–100						
D	4	65.0 (6.3)	100	100–100	100	100–100	100	100–100						
E	4	45.9 (16.1)	99.4	97.5–100	99.4	97.5–100	96.2	92.3–100						
F	4	50.2 (28.3)	100	100–100	100	100–100	100	100–100						
J	4	59.8 (2.6)	93.8	82.5–100	98.1	92.5–100	96.3	85.0–100						
***3–6 months***	***28***	***61.3 (16.2)***	***99.0***	***82.5–100***	***99.6***	***92.5–100***	***98.9***	***85.0–100***						
K	4	37.1 (11.3)	100	100–100	100	100–100	99.4	97.5–100	94.1	85.9–100	98.4	96.0–100	100.0	100–100
L	4	46.5 (8.7)	95.6	85.0–100	96.9	87.5–100	93.8	87.5–100	90.4	82.5–93.3	98.2	97.5–100	73.7	61.9–95.6
M	4	29.4 (28.1)	97.5	90.0–100	99.4	97.5–100	100	100–100	95.2	90.2–100	97.6	95.0–100	95.7	90.0–100
N	4	52.8 (8.4)	91.1	64.3–100	96.4	85.7–100	92.8	78.6–100	92.6	85.7–97.4	91.7	74.4–100	86.6	67.7–100
P	4	40.3 (20.8)	90.0	60.0–100	91.9	77.5–100	86.3	77.5–100	100	97.5–100	98.1	92.5–100	83.7	66.7–97.6
Q	4	37.5 (20.1)	89.6	63.4–100	98.7	97.6–100	94.5	82.9–100	96.3	95.0–97.6	97.5	95.0–100	92.2	83.4–97.6
R	4	47.2 (31.6)	86.3	50.0–100	93.8	75.0–100	91.7	80.0–100	89.7	78.7–97.5	96.6	91.5–100	96.5	88.8–100
S	4	40.5 (39.7)	78.4	25.6–97.6	93.7	82.1–100	83.3	33.3–100	89.0	70.0–97.7	94.8	84.2–100	93.2	80.6–100
T	4	51.9 (23.8)	90.1	67.5–100	93.2	75.0–100	96.9	87.5–100	93.6	87.8–100	98.1	92.3–100	90.2	73.7–100
U	4	37.6 (17.4)	87.6	70.0–100	95.6	87.5–100	88.2	72.5–100	83.8	50.0–97.6	92.8	80.0–100	91.9	79.0–97.9
J	4	49.5 (21.8)	100	100–100	100	100–100	100	100–100	89.2	59.5–100	94.1	76.3–100	35.3	5.1–42.3
***14–20 months***	***44***	***42.8 (21.4)***	***91.4***	***25.6–100***	***96.3***	***75.0–100***	***93.3***	***33.3–100***	***92.2***	***50.0–100***	***96.2***	***74.4–100***	***85.4***	***5.1–100***
K	4	35.8 (11.1)	100	100–100	100	100–100	98.6	94.3–100	93.8	85.0–100	96.9	90.0–100	61.3	30.0–90.0
L	4	49.8 (6.4)	100	100–100	100	100–100	97.4	94.7–100	90.0	70.0–100	96.9	92.5–100	54.4	45.0–60.0
M	4	73.6 17.7)	100	100–100	100	100–100	97.4	94.7–100	92.8	86.8–97.4	96.7	92.1–100	71.6	62.2–89.2
N	4	34.0 (18.4)	97.5	92.5–100	99.4	97.5–100	88.1	75.0–100	71.7	21.6–95.0	84.9	57.9–100	32.4	15.4–52.5
P	4	47.1 (20.5)	100	100–100	100	100–100	90.1	79.0–100	94.1	86.8–100	95.4	89.5–100	34.5	5.4–56.8
Q	4	43.8 (17.7)	100	100–100	100	100–100	93.9	91.9–94.6	91.3	87.5–97.5	88.8	82.5–95.0	56.9	52.5–62.5
R	4	33.5 (22.4)	100	100–100	100	100–100	89.2	77.1–100	75.0	57.5–92.5	78.8	55.0–95.0	28.1	0.0–50.0
S	4	36.9 (8.2)	98.8	95.0–100	100	100–100	96.6	89.2–100	59.4	32.5–75.0	63.1	35.0–77.5	38.8	15.0–85.0
T	4	37.7 (18.9)	88.1	57.5–100	89.4	62.5–100	89.6	68.4–100	67.5	25.0–92.5	70.6	30.0–100	23.8	12.5–37.5
U	4	58.4 17.1)	100	100–100	100	100–100	100	100–100	98.8	97.5–100	100	100–100	70.6	62.5–77.5
***26–32 months***	***40***	***45.1 (19.1)***	***98.4***	***57.5–100***	***98.9***	***62.5–100***	***94.1***	***68.4–100***	***83.4***	***21.6–100***	***87.2***	***30.0–100***	***47.2***	***0.0–90.0***

**Figure 6:**
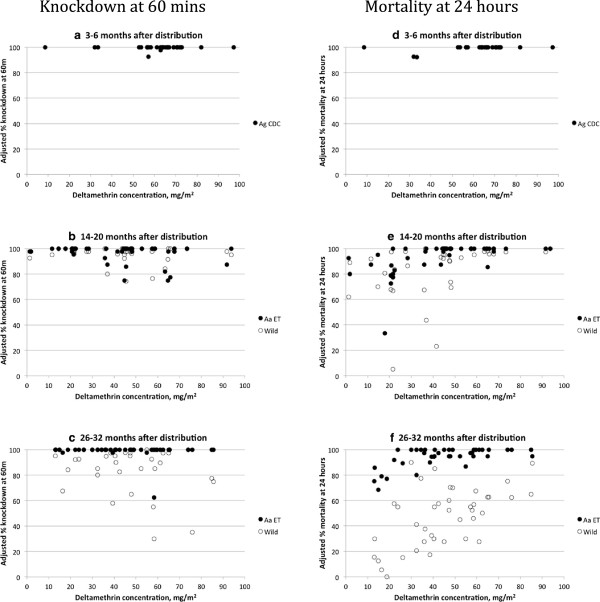
**Relationship between deltamethrin concentration and mortality in susceptible and wild caught mosquitoes by net bioassay.** Charts a, b, c: kd60 at three time periods, in laboratory strains (black dots) and wild caught moquitoes (white dots) Charts d, e, f: 24-hour mortality in susceptible mosquitoes from laboratory strains (black dots) and in wild caught mosquitoes (white dots). Ag CDC = *Anopheles gambiae* Kisumu strain tested at CDC Atlanta GA; Aa ET = *Anopheles arabiensis* Nazareth colony tested in Ethiopia; Wild = adults reared from larvae collected in Sodere, Oromia province, Ethiopia in November 2008 and 2009.

The knockdown data at 30 and 60 minutes for the bioassayed nets are presented as mean percentage knockdown and range in Table 2 and individual kd60 results per net are presented in Figure 6a-c. After three to six months, the average knockdown was 99.0% (range 82.5 to 100%) at 30 minutes and 99.6% (range 92.5 to 100%) knockdown at 60 minutes (kd60) with the susceptible mosquitoes. All but one of the 28 nets (96.4%) had > =95% knockdown at 60 minutes. At 14-20 months, kd60 averaged 96.3% (range 75 to 100%) and 96.2% (74.4 to 100%) with the susceptible Nazareth *An. arabiensis* and wild caught mosquitoes respectively. In these two groups, 81.8% (n = 44) and 84.1% (N = 40) of the bioassayed nets caused > =95% kd60 in the susceptible and resistant mosquitoes respectively. At 26-32 months, average kd60 was 98.9% (range 62.6-100%) for susceptible and 87.2% (range 30.0-100%) for wild-caught mosquitoes; the percentages of nets meeting the >95% kd60 criterion was 97.5% for susceptible mosquitoes but only 50.0% for the wild caught mosquitoes.

The mortality estimates at 24 hours after exposure observed for each net sample tested by bioassay are shown in Figures 6d-f. After three to six months, 25 of the 28 tested nets (89%) killed 100% of mosquitoes exposed for three minutes. For the remaining three nets, the lowest mortality observed at 24 hours was 85% (Figure 6d); therefore all 28 nets passed the threshold of > =80% mortality. The average mortality of 44 nets at 14-20 months with the susceptible Nazareth strain was 93.3% (range 89.6 to 97.0%) and with wild caught mosquitoes it was 85.4% (range 79.1 to 91.7%) (Figure 6e). The percentage of nets (N = 44) passing the >80% mortality cutoff was 90.9% and 72.3% with susceptible and resistant mosquitoes respectively. At 26-32 months the average mortality on 40 nets with the susceptible Nazareth strain was 94.1% (range 91.5 to 96.7%) and with wild caught mosquitoes was 47.2% (range 39.8 to 54.7%) (Figure 6f). The percentage of nets (N = 40) passing the >80% mortality cutoff was 90.0% and 7.5% with susceptible and resistant mosquitoes respcectively. The drop in mortality with the wild caught mosquitoes from 14-20 to 26-32 months can be seen in Figures 6e and f.

**Figure 7:**
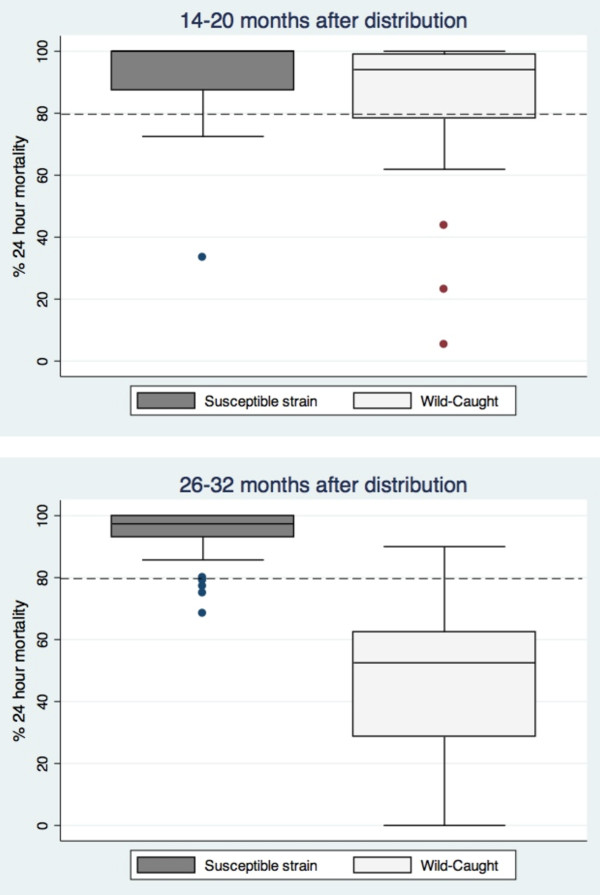
**Median 24-hour mortality with samples of bioassayed nets collected after 14-20 and 26-32 months.** Nets were tested in bioassays with both susceptible and wild caught mosquitoes. N = 28 for three to six month nets; N = 44 for 14-20 month nets; N = 40 for 26-32 month nets. The plots show median, 25^th^ and 75^th^ percentile (box) and fifth and 95% percentile (whisker) of the per cent mortality. Susceptible strain = *Anopheles arabiensis* Nazareth colony tested in Ethiopia; Wild-caught = adults reared from larvae collected in Sodere, Oromia province, Ethiopia in November 2008 and 2009 (at 14-20 months and 26-32 months respectively).

The drop in median mortality of wild caught mosquitoes on bioassayed nets between the 14-20 months and 26-32 month collections (from 85.4 to 52.5%, Figure 7) was statistically significant by the Mann Whitney non-parametric test (z = 6.398, p < 0.001). There was no change in median mortality between time periods in the susceptible strain.

There was not complete agreement between the two WHOPES recommended cutoffs for definition of an acceptable net (kd60 > =95% and mortality at 24 hours > =80%). For nets tested with susceptible mosquitoes (N = 112), taking the mortality > =80% cutoff as the gold standard, the sensitivity for using kd60 was 95.2% and specificity was 62.5%. For resistant mosquitoes, sensitivity was 89.2% but specificity 49.0%. In other words there were significant numbers of net samples where the kd60 suggested net effectiveness that was not borne out by the mortality results when knocked-down mosquitoes later recovered. Hence the more conservative estimate of mortality was used here.

**Figure 8:**
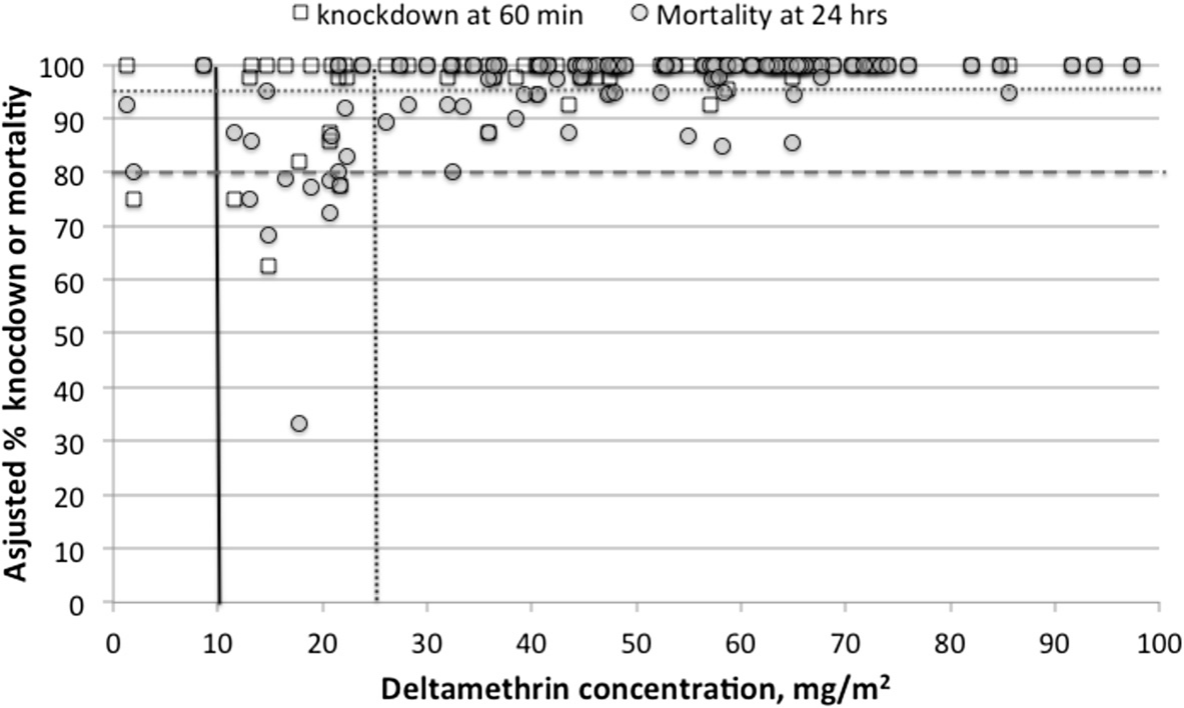
**Relationship between insecticide concentration on nets and knockdown/mortality in bioassay with susceptible mosquito strains.** White squares = kd60; grey circles = 24 hr mortality. Nets from all time periods of collection are included. Vertical solid line: minimum effective concentration of deltamethrin; dotted vertical line: optimum concentration; dotted horizontal line: WHOPES recommended standard of 95% for kd60; dashed horizontal line: WHOPES recommended standard of 80% for 24-hour mortality.

In order to shed light on the relationship between insecticide concentration and mortality by bioassay, Figure 8 plots the insecticide concentration results for each net tested by bioassay against the kd60 and 24 hour mortality it produced in susceptible colony mosquitoes (at any time period). Figure 8 illustrates that for knockdown, 91.1% (102/112) of the nets caused above 95% kd60, but those failing included 3 nets with above 25 mg/m^2^ estimated deltamethrin concentration, suggesting that >95% kd60 may be too stringent a criterion for an 'effective’ net. The great majority of nets tested from those collected at all time periods (104/112 or 92.9%) were above the threshold of 80% 24-hour mortality, and this included the majority of the nets with <10 and <25 mg/m^2^. However, the chart does provide some justification for the threshold of 25 mg/m^2^ for 'optimal’ concentration, since all nets above that concentration had > =80% mortality. However, so did 60% of the 20 nets below that threshold, including all three of the nets with <10 mg/m^2^. Thus 24 hr mortality is a more sensitive but less specific method of estimating deltamethrin concentration.

## Discussion

In this study, as noted by Wills *et al*. [9], net evaluation began very shortly after distribution and continued up to 32 months. Average insecticide levels declined by about one third over the first 14-20 months of use, but remained relatively stable after that period. Despite this decline, in common with some other investigators [11], it was found that insecticide persisted on the majority of the nets at above minimum effective concentration for the whole time period. However in contrast, in Ghana, after 38 months in use, it was observed that only 14% of nets retained sufficient active insecticide concentration to be effective [10]. However, both these studies suffer from the limitation that net attrition was not measured i.e. nets that had been destroyed and no longer in use were not available for sampling. If LLIN monitoring (including loss by attrition) is planned for a distribution campaign, it is vital to arrange for some unique identifier to be on the nets before being distributed in order to confirm their identities during subsequent collection. If nets are shipped internationally, it is essential that the nets be treated to kill bedbugs before shipment to regions where these insects are not endemic, e g, by freezing at -80°C for three days to destroy any infestation without damaging the net for subsequent analysis.

Evaluation of the relationship between deltamethrin concentration on nets tested by HPLC and XRF in this study showed them to be closely correlated, with a regression coefficient of 0.94, and r^2^ of over 90%. After three to six months in use, the estimated mean amount of deltamethrin per net was estimated to be higher than was reported by the manufacturer for the batches distributed in this study (59.8 mg/m^2^), most likely due to the analytical method used by the manufacturer which measures only the *S* isomer of the deltamethrin molecule. The XRF and HPLC methods used in this paper are not capable of distinguishing the *S* isomer from the biologically inactive *R* isomer (present as a minor component).

Given that XRF measures bromine atoms in the deltamethrin molecule, if the molecule disintegrates or epimerizes (changes to an inactive form) during use, the bromine may still remain. Bromine would only be lost if the deltamethrin was completely washed off or vaporized. Since deltamethrin is known to epimerize to the inactive R-isomer by exposure to heat [19] there is the possibility that this would cause bias over time in the estimation of deltamethrin by the XRF method. The extent of this bias is unknown, and may also be affected by net colour. This should be investigated in further evaluation of the XRF method against HPLC or gas chomatography over longer time periods and with different nets (including colours other than blue) than was done here, with attention also being paid to the concentration likely available on the surface of the nets.

Despite these discrepancies, the XRF results are useful for tracking the rate of deltamethrin loss over time, and for comparing levels for nets collected from different locations. The XRF method was found to be a rapid method for determining average deltamethrin levels on large numbers of nets, although it does require the use of an expensive (but portable) machine. However, the fact that XRF cannot distinguish the *S* (active) and *R* (inactive) isomers of deltamethrin and may overestimate slightly the actual concentration of active ingredient suggests caution in interpreting absolute values for deltamethrin.

At distribution, PermaNet 2 nets are stated by the manufacturer to have an average concentration of 55 mg/m^2^ of deltamethrin. A deltamethrin level of 10 mg/m^2^ is commonly proposed as the minimum effective concentration, while 15 mg/m^2^ is suggested by Kilian *et al.*[17] and 25 mg/m^2^ by Green *et al*. [18] as optimal. This study provides support for the threshold of 25 mg/m^2^ as being a meaningful cut-off above which all nets appear to be effective at WHOPES-recommended mortality cut-off of > = 80%.

Insecticide on the nets estimated by XRF in this study shows very good retention, with only 8/200 (4.0%) nets falling below the minimum effective concentration of 10 mg/m^2^ and 41/200 (20.5%) below the optimal concentration (<25 mg/m^2^) by 32 months after distribution. Bioassays with susceptible *Anopheles* confirmed the presence of sufficient insecticide to cause sufficient mortality at 24 hours on more than 90% of the nets at all time periods. While this study only continued for about 2.5 years (26-32 months), the results concur with the findings of Kilian *et al.*[17] that PermaNet 2 generally show very good effective insecticide retention through their physical life. However, like the physical deterioration results [9], the loss rate of insecticide appears to vary by collection site. The effects of household and site level variables will be explored separately.

The ability of nets with sufficient deltamethrin concentration to kill mosquitoes also depends on level of resistance to this insecticide in the wild population. Most wild caught mosquitoes from one site at Sodere (near Nazareth/Adama in Oromia province) were killed by the nets after up to 14-20 months of use. This is consistent with the findings of Balkew *et al.*[20] who observed that *An. arabiensis* mosquitoes collected in Sodere between Nov 2005 and April 2007 (just before the commencement of the current study) were 99.2% susceptible to deltamethrin as measured by 24 hr mortality, although time to knockdown was surprisingly high (suggesting partial resistance) and similar to knockdown times observed in other areas of Ethiopia where the mosquitoes showed partial resistance to pyrethroids. However, by the third collection in the current study in 2009 after 26-32 months use, there was significantly lower mortality (i.e. resistance) in the wild caught mosquitoes, suggesting the rapid development of resistance to deltamethrin in the wild population around the capture site of Sodere and perhaps elsewhere. This confirms the results of Yewhalew *et al*. [21] who observed that PermaNet 2.0 were only 47.4 to 77.8% effective at killing wild caught mosquitoes from four sites in Ethiopia, using cone bioassays on new nets.

A limitation of the results is that the wild caught mosquitoes were not identified by PCR to species (*An. gambiae* or *An. arabiensis*) in either year, and it is therefore not completely certain that they were *An. arabiensis*. However, previous studies from the same location have only found *An. arabiensis* in larval or pupal collections [20]. Testing and identification of wild caught mosquitoes from additional sites on a routine basis is recommended, but ideally in conjunction with colony mosquitoes so that the nets can be assessed as well as the wild populations. Fettene *et al*. [22] tested nets collected from households in Buie and Fentale districts of Ethiopia by bioassay with a susceptible *An. arabiensis* strain. Although knock-down was high, they observed 24-hour mortality (72 and 67%, respectively) lower than observed here. They state that this low mortality may be due to the age of the nets (some were distributed more than three years before collection), repeated washing or build-up of dirt on the nets [22]. Nevertheless, given that DDT resistance is widespread, the problem of resistance to pyrethroids in Ethiopia (either *kdr* or other suspected mechanisms) and assessment of its status throughout the country must be urgently addressed [20].

Although this study set out to generate data describing the deterioration of LLINs in actual use, the determination of at what point a net, or a batch of nets, has deteriorated sufficiently to warrant replacement remains a matter of judgement. It appears from the previous paper on these net samples [9] that physical damage is potentially more important and rapid than loss of insecticide. However, one of the original motivations for treating bed nets with insecticide was to maintain efficacy even when nets are compromised by holes. In fact, the evaluation of LLINs using the WHOPES Phase II experimental hut testing protocol stipulates the use of nets having six 4 × 4 cm holes (hole area = 96 cm^2^) in order to simulate natural damage [3]. Several studies that addressed this issue [23-27] found that treated nets with holes (either intentionally cut or natural) significantly decreased house entering, blood feeding and biting by *Anopheles* or *Culex*, or increased mosquito mortality, suggesting that the insecticide compensates for most of the loss of protection otherwise caused by the holes. However, no definitive study or review exists that describes the loss of efficacy in protection from malaria as the number and size of holes increase, and the level of insecticide decreases. These studies illustrate the need for a more complete understanding of the relationship between net condition, care and protective efficacy. This understanding is vital for providing meaningful guidance to planners engaged in vector control programmes and net replacement campaigns.

## Conclusions

The current study provides a description of the rate of chemical deterioration of PermaNet 2.0 LLINs, distributed in Ethiopia with support from The Carter Center at known times starting in 2007. Insecticide levels estimated by a convenient method of XRF sprectroscopy were significantly diminished by 32 months of use, but 96% of nets still had estimated concentrations greater than the minimum (10 mg/m^2^), and 79.5% of nets still had concentrations above the optimal level of 25 mg/m^2^. Since the XRF method measures bromine and cannot distinguish the inactive isomer of deltamethrin, it may overestimate slightly the true insecticide concentration. However, all nets tested with estimated concentrations of more than 25 mg/m^2^ were effective in killing more than 80% of susceptible mosquitoes by bioassay. Therefore, this study provides evidence for the development of resistance to deltamethrin in the population of *An. arabiensis* in Sodere, Ethiopia by 2009, since there was reduced efficacy of nets that had above-optimal concentration of insecticide.

Results varied by location, and it is clear that many extrinsic factors impact LLIN deterioration. Therefore, agencies considering follow-up distribution campaigns should monitor LLIN performance after distribution and actual use using longitudinal studies of individually identified nets, rather than rely on results extrapolated from laboratory or limited field studies, or on longevity claims by manufacturers. The median half-life of LLIN and the point at which they need to be replaced is still a matter of discussion and judgment. It is clear however that net deterioration is a continuous and gradual process, and that a blanket policy of replacement of all nets after three years is not necessarily appropriate. Given the good insecticide retention observed in this study, net durability appears to be determined more by physical deterioration than insecticide persistence. However, if resistance to pyrethroids including deltamethrin becomes widespread in Ethiopia, the duration of its persistence on nets becomes irrelevant, and this urgent problem must be addressed.

## Competing interests

The authors declare that they have no competing interests.

## Authors’ contributions

The study was planned by PME, PMG, SS, AWM, and TG. AW, SS, GYA, and MD collected nets from the field with assistance from EBS, TE and TG. SS, AW, GYA, and MD conducted the insecticide measurements and data management. GYA and AW did the bioassays. PMG, SS, AW, and AEP did data management and analysis. AWM did the mapping. PMG, SS and AW wrote the paper, which was reviewed by all authors. All authors read and approved the final manuscript.
